# LncRNA LINC01116 sponges miR-93-5p to promote cell invasion and migration in small cell lung cancer

**DOI:** 10.1186/s12890-020-01369-3

**Published:** 2021-02-03

**Authors:** Wenzhou Liu, Feihai Liang, Guangyu Yang, Lei Xian

**Affiliations:** Department of Thoracic and Cardiovascular Surgery, The Second Affiliated Hospital of Guangxi Medical University, No.166 Da Xuedong Road, Nanning City, Guangxi Province 530007 People’s Republic of China

**Keywords:** Small cell lung cancer, LINC01116, miR-93-5p, Survival, STAT3

## Abstract

**Background:**

LINC01116 is a recently identified oncogenic lncRNA in glioma. Differential expression analysis using the public gene expression analysis tool GEPIA revealed the upregulation of LINC01116 in lung cancer. We studied the functions of LINC01116 in small cell lung cancer (SCLC).

**Methods:**

The expression of LINC01116 in several types of cancer tissue and the paired non-tumor tissues was evaluated by GEPIA. The effects of the overexpression of LINC01116 and miR-93-5p on the expression of STAT3 were evaluated. The effects of the overexpression of LINC01116, miR-93-5p and STAT3 on SHP-77 cell behaviors were evaluated by Transwell assays.

**Results:**

LINC01116 was highly expressed in SCLC and predicted poor survival. In SCLC tissues, the expression of LINC01116 was positively correlated with STAT3. Bioinformatics analysis revealed that miR-93-5p may target LINC01116. Overexpression of LINC01116 increased STAT3 but did not affect the expression of miR-93-5p. Transwell assay showed that LINC01116 and STAT3 increased cell invasion and migration rates. MiR-93-5p played an suppressed cell behaviors and suppressed the role of LINC01116.

**Conclusion:**

Therefore, LINC01116 might upregulate STA3 by sponging miR-93-5p, thereby promoting cell invasion and migration in SCLC.

## Background

As a type of deadly tumor, small cell lung cancer (SCLC) accounts for about 1/6 of lung cancer [1]. Although SCLC is closely correlated with smoking, SCLC also affects never-smokers, especially females [2]. Current knowledge of the pathogenesis of SCLC is still limited [3]. Patients with SCLC are usually treated with platinum in combination with etoposide and topotecan, which are the first- and second-line drugs for this disease, respectively [4]. Several other drugs, such as doxorubicin, cyclophosphamide and vincristine, have also been developed to treat SCLC, while no significant improvement in the survival of patients was observed [5]. It is believed that there are no advances in the treatment of SCLC during the past several decades.

SCLC requires the involvement of molecular factors [6, 7]. Molecular facts involved in SCLC may also the targets for treatment [7] STAT3 regulates inflammation, immunity and metastasis in cancer [8, 9]. MiR-93-5p can target STAT3 to inhibit cancer metastasis [10]. Previous studies showed that LINC01116 promotes glioma, ovarian cancer and breast cancer [11–13]. Analysis of TCGA by GEPIA (http://gepia.cancer-pku.cn/) revealed the upregulation of LINC01116 in several types of lung cancer. In addition, LINC01116 and miR-93-5p were predicted to interact with each other. We then studied the crosstalk between LINC01116, miR-93-5p and STAT3 in SCLC.

## Methods

### SCLC patients

The present study included 62 SCLC patients (gender: 39 males and 23 females; age range: 43 to 67 years old; mean age: 55.3 ± 6.9 years old) who were selected from the 122 cases of SCLC admitted to the Second Affiliated Hospital of Guangxi Medical University (this hospital Ethics Committee approved this study) between May 2011 and May 2014. Inclusion criteria: (1) newly diagnosed SCLC cases; (2) confirmed by histopathological biopsy. Exclusion criteria: (1) any treatments within 3 months prior to admission; (2) recurrent cases; (3) any other clinical disorders were diagnosed. This study passed the review board of the Ethics Committee of the aforementioned hospital.

### Biopsy and specimens

During biopsy, SCLC (tumor) tissues and adjacent (2 cm around tumor) non-tumor lung tissues (control group) were obtained through biopsy.

### Staging, treatment and follow-up

Based on clinical findings, the 62 SCLC patients were staged into stage II (n = 18), III (n = 22) and IV (n = 22). According to patients’ conditions, surgical removal in combination with chemotherapy (n = 18) or radiotherapy (n = 10), or chemotherapy (n = 20) and radiotherapy (n = 14) alone were performed. From the day of admission, a 5-year follow-up study was carried out. Patients died of other diseases or accidents were excluded. Patients failed to complete the follow-up were also excluded.

### SCLC cell line and transfection

Human SCLC cell lines SHP-77 and DMS 53 (ATCC, USA) were used in this study. The cell culture medium was a mixture of 10% FBS and 90% RPMI-1640 medium. Under the conditions of 37 °C, 95% humidity and 5% CO_2,_ cells were cultivated to reach 80% confluence, followed by transient transfections. Cells were transfected with pcDNA3.1-LINC01116 expression vector, pcDNA3.1-STAT3 expression vecto or miR-93-5p mimic (Invitrogen) using lipofectamine 2000 (Sigma-Aldrich). NC and C experiments were also included.

### RNA extractions and qPCR

Total RNAs were extracted from 10^5^ SHP-77 cells (harvested at 48 h post-transfection) or 0.01 g tissue samples using RNAzol (Sigma-Aldrich). RNA precipitation was performed using 85% ethanol to retain miRNAs. All RNA samples were digested by DNase I at 37 °C for 2 h to remove genomic DNAs. Reverse transcription was performed using the MMLV Reverse Transcriptase 1st-Strand cDNA Synthesis Kit (Lucigen). All qPCR reactions were prepared using the QuantiTect SYBR Green PCR Kit (Qiagen). The expression levels of LINC01116 and STAT3 mRNA were analyzed with internal control GAPDH. Mature miR-93-5p (not precursor) expression was analyzed using All-in-One™ miRNA qRT-PCR Detection Kit (Genecopoeia).

### Western blot

RIPA and BCA assay (Invitrogen) were applied for protein extraction and quantification, respectively. After denaturation, proteins were separated using 10% SDS-PAGE gels. After gel transfer (PVDF membranes) and blocking, incubation with primary and secondary antibodies was performed. Primary antibodies were rabbit anti-GAPDH (1:1800, ab22555, Abcam) and-STAT3 (1:1800, ab226942, Abcam). of the secondary antibody was goat HRP (IgG) (1:2000; ab6721; Abcam). Signals were developed using ECL (Thermo Fisher Scientific). Data was analyzed using Image J v1.46 softwares.

### Transwell assays

Transwell assays were used to measure invasion and migration rates of SHP-77 and DMS 53 cells at 48 h post-transfection. To prepare cell suspensions, 3 × 10^4^ cells were mixed with 1 ml non-serum RPMI-1640 Medium. Cells were transferred to upper Transwell assay. The lower chamber was added with 20% FBS. Matrigel (250 μg/ml, Millipore, USA)—coated membrane was only used in invasion assay. After cell culture for 12 h, 0.1% crystal violet (Sigma-Aldrich, USA) was used to stain cells at 24 °C for 20 min. Cell invasion and migration were observed under a light microscope. Cells were counted using Image J v1.46 software.

### Statistical analysis

Paired tissues were compared by paired t test. Cell transfection groups were compared by ANOVA Tukey’s test. To perform survival analysis, the 62 patients were grouped into high and low LINC01116 level groups (n = 31, cutoff value = the median expression level of LINC01116 in SCLC). Survival curves were plotted and log-rank test was used for the comparison. *p* < 0.05 was considered as statistically significant.

## Results

### Upregulation of LINC01116 predicted poor survival of SCLC patients

**Fig. 1:**
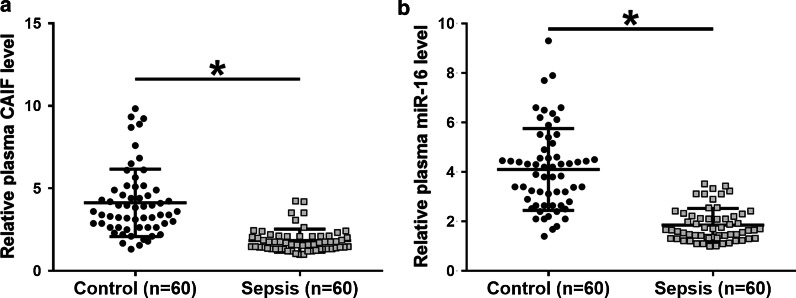
Upregulation of LINC01116 predicted poor survival of SCLC patients. Expression levels of LINC01116 in both SCLC and non-tumor tissues from the 62 SCLC patients were measured by qPCR and paired test, respectively (**a**). Mean values of 3 replicates were presented. To perform survival analysis, the 62 patients were grouped into high and low LINC01116 level groups (n = 31) with the median expression level of LINC01116 in SCLC as cutoff value. K-M plotter was used to plot survival curves and log-rank test was used for the comparison (**b**)

The expression of LINC01116 in several types of cancer tissue and the paired non-tumor tissues was evaluated by GEPIA. The results revealed that LINC01116 was upregulated in several types of lung cancer (see results: http://gepia.cancer-pku.cn/detail.php?gene=LINC01116). In this study, the expression levels of LINC01116 in both SCLC and non-tumor tissues from the 62 SCLC patients were detected by qPCR. The expression levels of LINC01116 were significantly increased in SCLC tissues (Fig. 1a, *p* < 0.05). In addition, compared with patients in the low LINC01116 level group, patients in the high LINC01116 level group showed significantly lower overall survival rate (Fig. 1b, *p* < 0.05). The overall survival time of patients in the low LINC01116 level group ranged from 10 to more than 60 months, and this range in the high LINC01116 level group was 5 to more than 60 months. Therefore, measurement of the expression levels of LINC01116 before therapy might assist the prognosis of SCLC.

### MiR-93-5p may bind to LINC01116 but did not regulate its expression

**Fig. 2:**
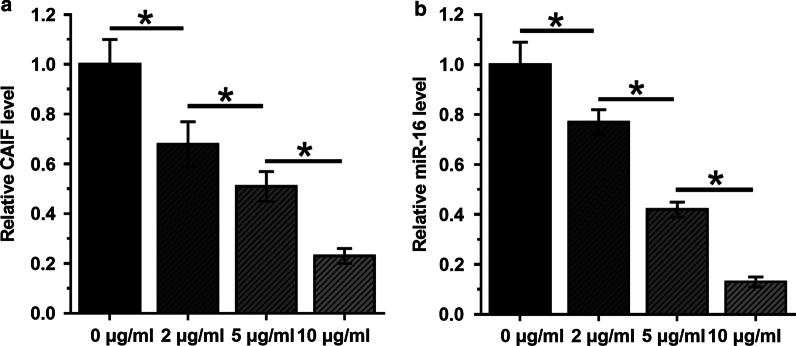
MiR-93-5p may bind to LINC01116 but did not regulate its expression. RNA interaction prediction using IntaRNA (http://rna.informatik.uni-freiburg.de/IntaRNA/Input.jsp) showed that miR-93-5p may form base pairing with LINC01116 (**a**). Overexpression of miR-93-5p and STAT3 was confirmed by qPCR at 48 h post-transfection (**b**). The interaction between miR-93-5p and STAT3 was analyzed by qPCR (**c**). Mean values of 3 replicates were presented, *, *p* < 0.05

RNA interaction analysis using IntaRNA showed that miR-93-5p could form base pairing with LINC01116 (Fig. 2a). To further detect the interactions between miR-93-5p and LINC01116, SHP-77 cells were overexpressed with miR-93-5p or LINC01116 expression. The expression of miR-93-5p and STAT3 was significantly upregulated (Fig. 2b, *p* < 0.05). However, overexpression of miR-93-5p and LINC01116 did not affect the expression of each other (Fig. 2c). Therefore, LINC01116 was unlikely a target of miR-93-5p.

### LINC01116 was positively correlated with STAT3 in SCLC

**Fig. 3:**
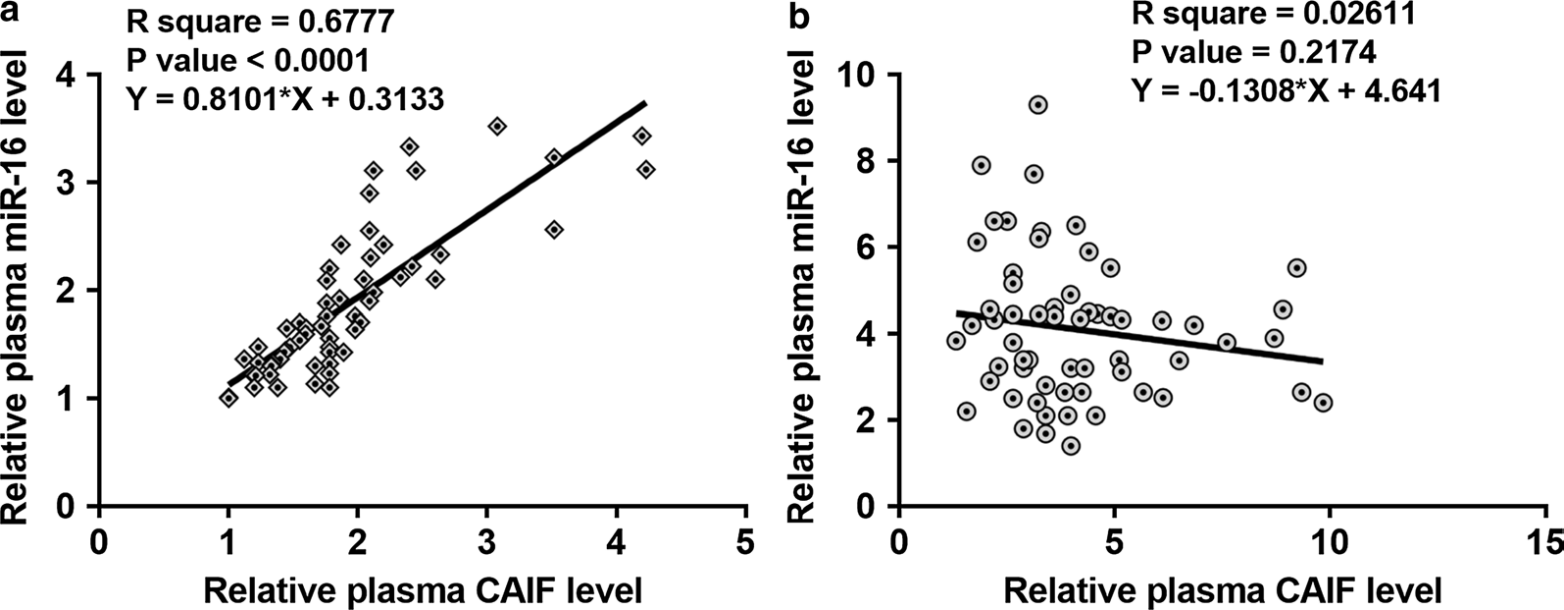
LINC01116 was positively correlated with STAT3 in SCLC. STAT3 is a direct target of miR-93-5p. Expression levels of STAT3 mRNA in both SCLC and non-tumor tissues from the 62 SCLC patients were measured and compared by performing qPCR and paired t test, respectively (**a**). Mean values of 3 replicates were presented. Correlation between LINC01116 and STAT3 mRNA across SCLC tissues was analyzed by Pearson’s correlation coefficient (**b**)

RT-qPCR assays illustrated that expression levels of STAT3 were significantly increased in SCLC tissues (Fig. 3a, *p* < 0.05). Correlation between LINC01116 and STAT3 mRNA across SCLC tissues was analyzed by Pearson’s correlation coefficient. It was observed that LINC01116 was positively and significantly correlated with STAT3 in SCLC (Fig. 3b). Therefore, LINC01116 might interact with STAT3 through miR-93-5p.

### Overexpression of LINC01116 led to upregulated STAT3 through miR-93-5p in SHP-77 cells

**Fig. 4:**
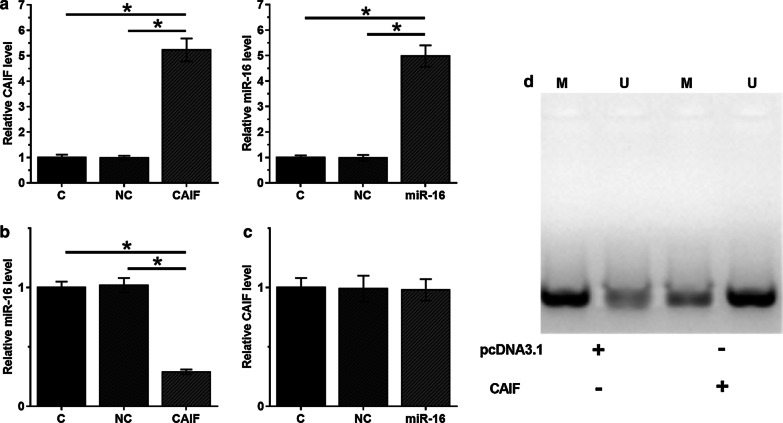
Overexpression of LINC01116 led to upregulated STAT3 through miR-93-5p. The effects of overexpressing LINC01116 and miR-93-5p on the expression of STAT3 were evaluated by both qPCR (**a**) and western blot (**b**). Mean values of 3 replicates were presented, *, *p* < 0.05

The roleof LINC01116 and miR-93-5p in regulating STAT3 expression were evaluated by qPCR (Fig. 4a) and western blot (Fig. 4b). Overexpression of LINC01116 led to upregulated STAT3 (*p* < 0.05). In addition, miR-93-5p played an opposite role and attenuated the effects of overexpressing LINC01116 (*p* < 0.05). Therefore, LINC01116 could upregulate STAT3 possibly by sponging miR-93-5p.

### LINC01116 promoted SHP-77 and DMS 53 cell behaviors through miR-93-5p and STAT3

**Fig. 5:**
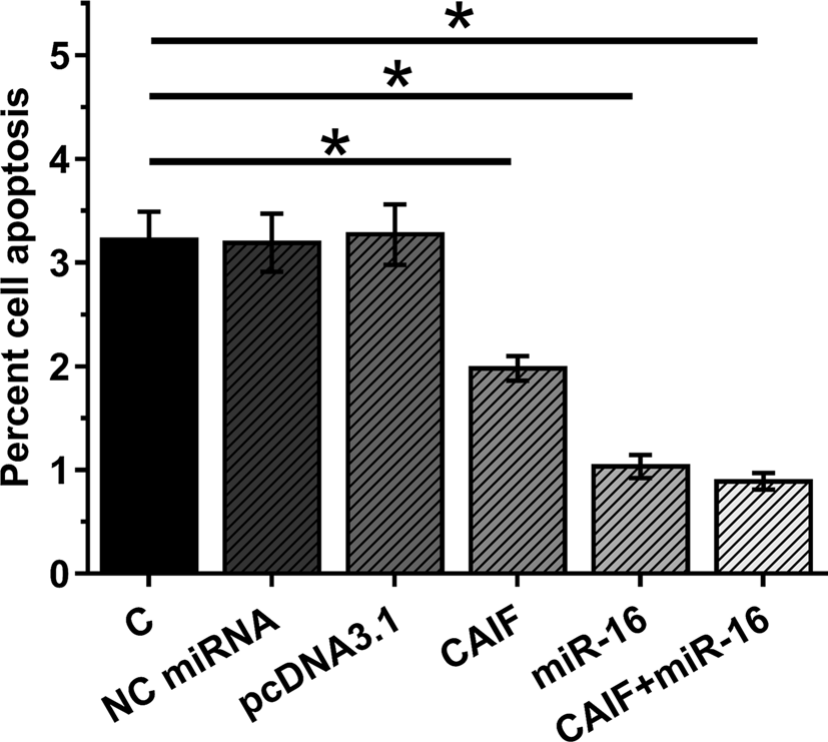
LINC01116 promoted SHP-77 cell invasion and migration through miR-93-5p and STAT3. The effects of overexpression of LINC01116, miR-93-5p and STAT3 on SHP-77 cell invasion (**a**) and migration (**b**) analyzed by Transwell assays. Mean values of 3 replicates were presented, *, *p* < 0.05

The effects of the overexpression of LINC01116, miR-93-5p and STAT3 on SHP-77 cell invasion (Fig. 5a) and migration (Fig. 5b) were evaluated by Transwell assays. Overexpression of LINC01116 and STAT3 led to increased cell invasion and migration rate of SHP-77 cells (*p* < 0.05). Overexpression of miR-93-5p played an opposite role and attenuated the effects of the overexpression of LINC01116 (*p* < 0.05). In addition, DMS 53 cell line was used to repeat the Transwell assay. Similarly, overexpression of LINC01116 and STAT3 increased cell invasion and migration rates of DMS 53 cells (*p* < 0.05). MiR-93-5p overexpression suppressed cell behaviors and attenuated the effects of overexpressing LINC01116 (*p* < 0.05). Therefore, LINC01116 could promote SCLC cell invasion and migration possibly by sponging miR-93-5p.

## Discussion

The functions of LINC01116 in SCLC were investigated in this study. We found that LINC01116 was upregulated in SCLC and may sponge miR-93-5p to promote the invasion and migration of SCLC cells. In addition, high expression levels of LINC01116 in SCLC tissues predicted poor survival of SCLC patients.

The functions of LINC01116 have been characterized in several types of cancer [11–13]. In glioma, LINC01116 is overexpressed and can target VEGFA to promote the tumorigenesis of glioma [11]. LINC01116 is also upregulated in epithelial ovarian cancer and can inhibit the apoptosis of cancer cell to promote cancer progression [12]. Another recent study reported that LINC01116 was upregulate in breast cancer and reduced the effects of overexpressing miR-145 on the expression of ESR1 to promote cancer progression [13]. All previous studies suggested the oncogenic functions of LINC01116. To the best of our knowledge, this is the first study to report the upregulation of LINC01116 in SCLC. In addition, overexpression of LINC01116 led to increased rates of SCLC cell invasion and migration. Our data suggested the oncogenic role of LINC01116 in SCLC.

MiR-93-5p plays different roles in different types of cancer [14, 15]. MiR-93 promotes cancer progression in gastric cancer by inactivating the Hippo signaling pathway [14]. However, miR-93 interacts with WNK1 to inhibit the invasive potential of cancer cells in triple-negative breast cancer [15]. A recent study found that miR-93-5p could target STAT3 to inhibit epithelial-mesenchymal transition in breast cancer [10]. In the present study, we observed downregulated expression of STAT3 in SCLC cells after the overexpression of miR-93-5-p. In addition, overexpression of miR-93-5-p led to suppressed cancer cell invasion and migration. Therefore, miR-93-5-p may target STAT3 and suppress cancer cell invasion and migration in SCLC.

We found that miR-93-5-p may bind to LINC01116, while overexpression experiments revealed no interactions between them. In addition to severing as the targets of miRNAs, lncRNAs may sponge miRNAs to attenuate their effects on the expression of downstream genes [16, 17]. In this study, we observed upregulated STAT3 after the overexpression of LINC01116. Therefore, LINC01116 may sponge miR-93-5p to upregulate STAT3 in SCLC.

The role of lncRNAs as the spongy of miRNAs has been extensively studied [17, 18]. With an increased understanding of the regulations of multi-functional lncRNAs, novel anti-cancer approaches may be developed. Therefore, future studies are of great importance to investigate the interactions between lncRNAs and miRNAs at genome-wide level.

## Conclusions

In conclusion, LINC01116 is upregulated in SCLC and plays oncogenic roles, which are likely mediated by sponging miR-93-5p to upregulate STAT3.

## Data Availability

The analyzed data sets generated during the study are available from the corresponding author on reasonable request.

## Abbreviations

SCLC: Small cell lung cancer

## References

[1] Gazdar AF, Bunn PA, Minna JD (2017). Small-cell lung cancer: what we know, what we need to know and the path forward. Nat Rev Cancer.

[2] Pirie K, Peto R, Green J, Reeves GK, Beral V, Collaborators MWS (2016). Lung cancer in never smokers in the UK Million Women Study. Int J Cancer.

[3] Gazdar AF, Minna JD (2019). Small cell lung cancers made from scratch.

[4] Waqar SN, Morgensztern D (2017). Treatment advances in small cell lung cancer (SCLC). Pharmacol Ther.

[5] Koinis F, Kotsakis A, Georgoulias V (2016). Small cell lung cancer (SCLC): no treatment advances in recent years. Transl Lung Cancer Res.

[6] Wistuba II, Gazdar AF, Minna JD (2001). Molecular genetics of small cell lung carcinoma. Semin Oncol.

[7] Sumimoto H, Yamagata S, Shimizu A, Miyoshi H, Mizuguchi H, Hayakawa T, Kawakami Y (2005). Gene therapy for human small-cell lung carcinoma by inactivation of Skp-2 with virally mediated RNA interference. Gene Ther.

[8] Yu H, Pardoll D, Jove R (2009). STATs in cancer inflammation and immunity: a leading role for STAT3. Nat Rev Cancer.

[9] Kamran MZ, Patil P, Gude RP (2013). Role of STAT3 in cancer metastasis and translational advances. BioMed Res Int.

[10] Xiang Y, Liao X-H, Yu C-X, Yao A, Qin H, Li JP, Gu CJ (2017). MiR-93-5p inhibits the EMT of breast cancer cells via targeting MKL-1 and STAT3. Exp Cell Res.

[11] Ye J, Zhu J, Chen H, Qian J, Zhang L, Wan Z, Luo C (2020). A novel lncRNA-LINC01116 regulates tumorigenesis of glioma by targeting VEGFA. Int J Cancer.

[12] Fang Y, Huang Z, Li H, Tan W, Zhang Q, Wang L, Wu J (2018). LINC01116 promotes the progression of epithelial ovarian cancer via regulating cell apoptosis. Eur Rev Med Pharmacol Sci.

[13] Hu H, Chen Q, Ding S (2018). LncRNA LINC01116 competes with miR-145 for the regulation of ESR1 expression in breast cancer. Eur Rev Med Pharmacol Sci.

[14] Li L, Zhao J, Huang S, Wang Y, Zhu L, Cao Y, Deng J (2018). MiR-93-5p promotes gastric cancer-cell progression via inactivation of the Hippo signaling pathway. Gene.

[15] Shyamasundar S, Lim JP, Bay BH (2016). miR-93 inhibits the invasive potential of triple-negative breast cancer cells in vitro via protein kinase WNK1. Int J Oncol.

[16] Liang WC, Fu WM, Wong CW, Wang Y, Wang WM, Hu GX, Zhang JF (2015). The lncRNA H19 promotes epithelial to mesenchymal transition by functioning as miRNA sponges in colorectal cancer. Oncotarget.

[17] Thomson DW, Dinger ME (2016). Endogenous microRNA sponges: evidence and controversy. Nat Rev Genetics..

[18] Paraskevopoulou MD, Hatzigeorgiou AG (2016). Analyzing miRNA–lncRNA interactions. Methods Mol Biol.

